# Raman Spectroscopy of Oral *Candida* Species: Molecular-Scale Analyses, Chemometrics, and Barcode Identification

**DOI:** 10.3390/ijms23105359

**Published:** 2022-05-11

**Authors:** Giuseppe Pezzotti, Miyuki Kobara, Tamaki Nakaya, Hayata Imamura, Nao Miyamoto, Tetsuya Adachi, Toshiro Yamamoto, Narisato Kanamura, Eriko Ohgitani, Elia Marin, Wenliang Zhu, Ichiro Nishimura, Osam Mazda, Tetsuo Nakata, Koichi Makimura

**Affiliations:** 1 Ceramic Physics Laboratory, Kyoto Institute of Technology, Sakyo-ku, Matsugasaki, Kyoto 606-8585, Japan; timtam1213@outlook.jp (T.N.); hyt8888@outlook.jp (H.I.); elia-marin@kit.ac.jp (E.M.); wlzhu@kit.ac.jp (W.Z.); 2 Department of Immunology, Graduate School of Medical Science, Kyoto Prefectural University of Medicine, Kamigyo-ku, 465 Kajii-cho, Kyoto 602-8566, Japan; ohgitani@koto.kpu-m.ac.jp (E.O.); omazda@gmail.com (O.M.); 3 Department of Orthopedic Surgery, Tokyo Medical University, 6-7-1 Nishi-Shinjuku, Shinjuku-ku, Tokyo 160-0023, Japan; 4 Department of Dental Medicine, Graduate School of Medical Science, Kyoto Prefectural University of Medicine, Kamigyo-ku, Kyoto 602-8566, Japan; n-miya@koto.kpu-m.ac.jp (N.M.); t-adachi@koto.kpu-m.ac.jp (T.A.); yamamoto@koto.kpu-m.ac.jp (T.Y.); kanamura@koto.kpu-m.ac.jp (N.K.); 5 The Center for Advanced Medical Engineering and Informatics, Osaka University, 2-2 Yamadaoka, Suita 565-0854, Japan; 6 Division of Pathological Science, Department of Clinical Pharmacology, Kyoto Pharmaceutical University, 5 Misasagi Nakauchi-cho, Yamashina-ku, Kyoto 607-8414, Japan; kobara@mb.kyoto-phu.ac.jp (M.K.); nakata@mb.kyoto-phu.ac.jp (T.N.); 7 Division of Advanced Prosthodontics, The Jane and Jerry Weintraub Center for Reconstructive Biotechnology, UCLA School of Dentistry, Los Angeles, CA 90095, USA; inishimura@dentistry.ucla.edu; 8 Medical Mycology, Graduate School of Medicine, Teikyo University, Itabashi-ku, Tokyo 173-8605, Japan; makimura@med.teikyo-u.ac.jp

**Keywords:** Raman spectroscopy, *Candida*, chemometrics, barcode, molecular analyses

## Abstract

Oral candidiasis, a common opportunistic infection of the oral cavity, is mainly caused by the following four *Candida* species (in decreasing incidence rate): *Candida albicans*, *Candida glabrata*, *Candida tropicalis*, and *Candida krusei*. This study offers in-depth Raman spectroscopy analyses of these species and proposes procedures for an accurate and rapid identification of oral yeast species. We first obtained average spectra for different *Candida* species and systematically analyzed them in order to decode structural differences among species at the molecular scale. Then, we searched for a statistical validation through a chemometric method based on principal component analysis (PCA). This method was found only partially capable to mechanistically distinguish among *Candida* species. We thus proposed a new Raman barcoding approach based on an algorithm that converts spectrally deconvoluted Raman sub-bands into barcodes. Barcode-assisted Raman analyses could enable on-site identification in nearly real-time, thus implementing preventive oral control, enabling prompt selection of the most effective drug, and increasing the probability to interrupt disease transmission.

## 1. Introduction

*Candida* yeasts are commensal members in the normal microbiota of the oral cavity, but they can eventually switch from commensal to potent pathogens depending on several extrinsic factors, including age, flow rate and pH of saliva, type of diet, smoking, and use of dentures [1,2,3,4]. While age has been located as a main factor for increasing yeast population in normal oral microbiota [5], *Candida* infections could eventually develop on mouth mucous membranes and then spread into the bloodstream causing candidemia in immunocompromised hosts, namely, in patients suffering from acquired immune deficiency syndrome, leukemia or diabetes [6].

*Candida albicans* is the most frequent species in oral candidiasis of immunocompromised patients [6,7]. However, non-*albicans Candida* species have been recovered with increasing frequency, as a result of their decreased susceptibility to antifungal agents. Such a drug resistance scenario has developed during the last decades as a consequence of the growing use of random antifungal agents [8].

Although pathogenicity profile and sensitivity to specific antifungal agents both vary among different species [9,10], exposure to antifungal agents during candidiasis treatment provides a positive selection pressure for those non-*albicans* yeasts (e.g., *Candida glabrata* and *Candida krusei*) [11,12], which were reported to be intrinsically less sensitive to drugs than others species [13]. *Candida krusei* and *Candida glabrata* species have long been known for their decreased susceptibility to fluconazole [14]. A number of reports have also given clear accounts of the decreased susceptibility or, at least, the delayed killing kinetics of *Candida krusei* with respect to flucytosine, caspofungin, and amphotericin B [15,16,17,18]. These circumstances make this species an important indicator in monitoring the development of antifungal resistance [19,20]. Ishikawa et al. [21] reported about *Candida glabrata* isolates with reduced sensitivity during treatment with the echinocandin drug micafungin. Despite guidance indicating the use of echinocandins as the first-choice drug for the treatment of systemic infections of *Candida glabrata*, several independent publications reported about the resistance of this species to echinocandins [22,23,24]. *Candida tropicalis*, which has been associated with a high mortality rate and to the poorest prognosis among non-*albicans Candida* species [25], has also developed resistances to azole [26,27,28] and, in lower extent, to echinocandin [29]. Studies by Arthington-Skaggs et al. [30] and Rueda et al. [31] confirmed that *Candida tropicalis* is among the most azole-tolerant *Candida* species. When taking into consideration the limited number of antifungal drugs available to treat fungal infections, it becomes clear that the observed increasing trend in developing resistance against multiple antifungals poses a serious threat to patients with candidemia. This, in turn, calls for rapid and accurate methods of identification for *Candida* species, which could immediately allow the correct selection of the most effective antifungal agent, thus providing the patients with the best treatment possible since the initial drug prescription. Note that several methods are presently available for *Candida* identification in the clinical practice, which include: germ tube test, chlamydospore formation test, and sugar assimilation/fermentation tests. However, the germ tube test (the fastest method) only provides a limited range of speciation and is prone to false positive results. The latter two methods are labor intensive and time consuming (3~14 days). More recently established tests include CHROMagar^TM^, Candida Plus, API systems, and Vitek 2 ID system. These newer methods offer more rapid and cost-effective choices, but yet fail to locate all *Candida* yeast species. 

In this study, we examined the possibility to use Raman spectroscopy to identify *Candida* species distinctive to the oral cavity. Upon preliminary statistical validations of average spectra collected on *albicans* and non-*albicans* (i.e., *Candida glabrata*, *Candida krusei*, and *Candida tropicalis*) isolates, spectral differences among species were analyzed, linked to the respective cell molecular structures, and imaged in hyperspectral maps. While proving the high sensitivity of the Raman approach in locating oral *Candida* species, spectroscopic analyses based on a machine-learning algorithm specifically crafted for the Raman spectrum revealed direct links to the molecular structure of different species and provided chemical fingerprints allowing for their unequivocal identification. Chemometrics was then applied to probe for statistical validity and a *Raman barcode* approach employed to thoroughly classify different species. The analytical approaches followed in this study were in line with our recently published Raman studies of *Candida auris* clades [32,33], and bacteria and virus variants [34,35]. They also lie in the stream of Raman studies by other authors about the characterization of *Candida* isolates and related biofilms, which were performed with the aim to link the observed Raman spectra to genomic diversity [36,37,38,39]. While supporting already published analyses, data presented in this paper also offer new insights at the molecular scale and suggest a new approach regarding *Candida* species classification and patterning, as follows: (a) more detailed analyses were given of structural differences among oral *Candida* species including differences in glucan structures [40]; (b) visualization of such differences was achieved in microscopic chemical maps, and (c) a new classification method based on a barcode algorithm was proposed. When combined with Raman barcode classifications, Raman spectroscopy turns into a powerful molecular microscopy tool and could enable on-site label-free screening of *Candida* yeast cells. If properly developed with the buildup of a complete barcode library, the proposed Raman approach could represent the missing key in on-site candidiasis diagnostics not only in the oral cavity but also for candidemia in general.

## 2. Results

### 2.1. Morphological Observations of the Studied Species

**Figure 1:**
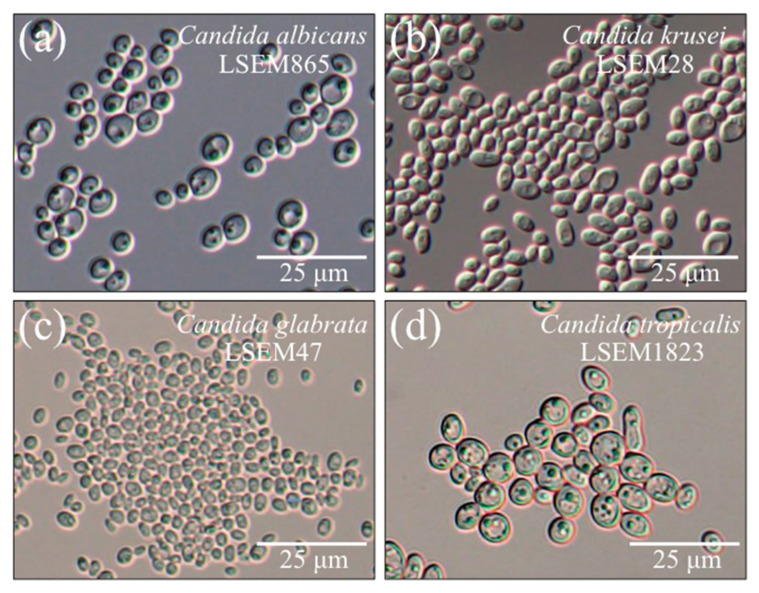
DIC micrographs of (**a**) *C. albicans*, (**b**) *C. krusei*, (**c**) *C. glabrata*, and (**d**) *C. tropicalis*.

Figure 1a–d show DIC micrographs of *C. albicans*, *C. krusei*, *C. glabrata*, and *C. tropicalis*, respectively. The cell size was measured and averaged upon measuring it on DIC micrographs. Among the studied species, cells of *C. tropicalis* appeared as the largest in size (~7.9 μm in average diameter), while those of *C. glabrata* were the smallest ones (2.9 μm in average diameter). Cells of both *C. albicans* and *C. krusei* showed intermediate sizes (5.6 and 4.9 μm in average diameter, respectively). All species showed fractions of cells with slightly elongated morphologies (maximum cell aspect ratio ~1.3). While the above morphological observations were in line with studies by other authors [41,42,43], it is generally recognized that mere morphological analyses are insufficient to characterize different *Candida* species and thus fail in predicting the strategy followed by pathogens to invade host and to evade its immune response. We shall probe here the possibility to refine vibrational analyses to an extent sufficient to enable univocal determination of different *Candida* species and, thus, to anticipate the expected strategies of host infection.

### 2.2. Raman Analyses of Different Candida Species

**Figure 2:**
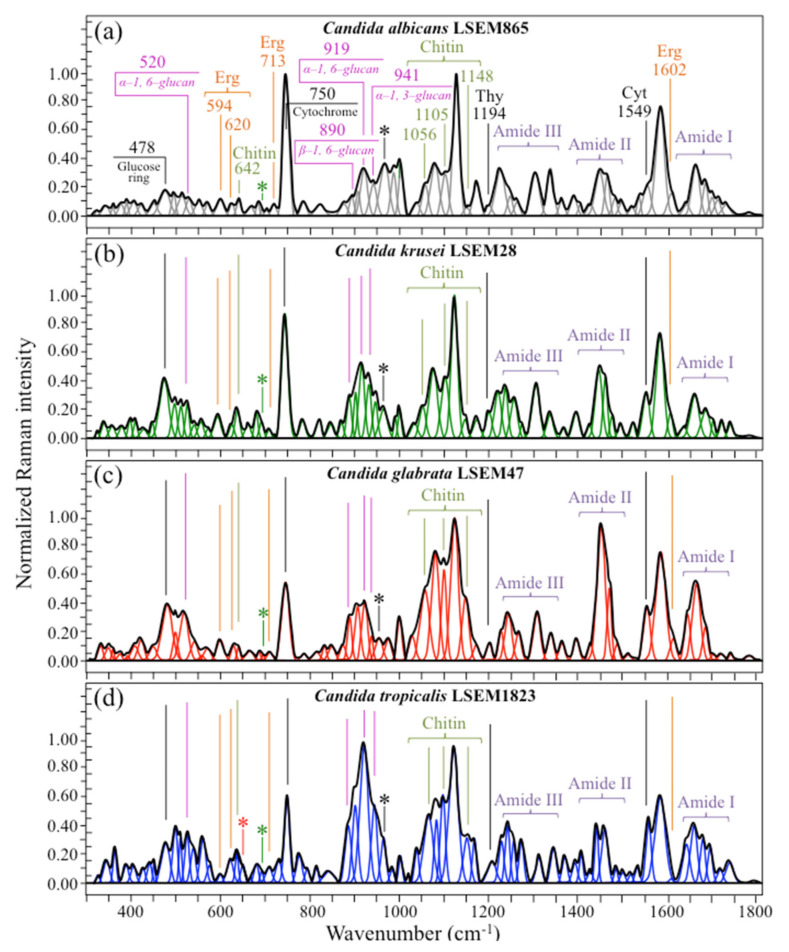
Raman spectra of (**a**) *C. albicans*, (**b**) *C. krusei*, (**c**) *C. glabrata*, and (**d**) *C. tropicalis*. Spectra are averages of 20 spectra collected on each sample with a 20× optical lens and with a spectral resolution better than 1 cm^−1^. The meaning of the asterisk marks is given in the main text.

Average Raman spectra of *C. albicans*, *C. krusei*, *C. glabrata*, *and C. tropicalis* are shown in Figure 2a–d, respectively. The spectra appeared morphologically similar, but clear differences among them could immediately be spotted even by the naked eye. This suggested that spectroscopy-based biochemical profiling could be possible for oral *Candida* species and encouraged us to attempt deeper analyses of Raman spectral differences, as described in the following.

The most striking difference detectable at a first glance was that the most intense band of the spectra differed among different species. In *C. albicans*, *C. krusei*, and *C. glabrata* (in Figure 2a–c, respectively), the most intense signal in the spectrum was the band at ~1123 cm^−1^, which is cumulatively related to C-C and C-O skeletal stretching in both polysaccharides [44]. Conversely, the most intense bands in the spectrum of *C. tropicalis* was located at ~919 cm^−1^, which is related to C-O-C stretching in α–1, 6–glucans (cf. Figure 2d; more details given later in this section). However, in *C. albicans*, another band with the same maximum intensity of the 1123 cm^−1^ band could be identified at ~750 cm^−1^ (cf. Figure 2a). This Raman band component, which is seen in all *Candida* species with variable intensity (cf. Figure 2a–d), is reported to be mainly contributed by breathing vibrations of the pyrrole ring [45], a structural feature present in a number of different biomolecules, including tryptophan, cytochrome, chlorophyll, and lactic acid. In the case of Raman spectrum of *Candida* yeasts, the 750 cm^−1^ band can mainly be assigned to cytochrome *c*. [46]. Cytochrome *c* is a redox-active heme protein, which is released by the mitochondria into the cytosol during apoptosis and undergoes redox state changes from Fe^2+^ to Fe^3+^ and vice versa; this molecule is a Raman-sensitive biomarker [47,48,49]. Its structure can undergo interconvertible switching from reduced heme Fe^2+^ to oxidized heme Fe^3+^ one. Main Raman bands characteristic of reduced cytochrome *c* are found at ~750, 1128, 1313, and 1585 cm^−1^, while a band at 1638 cm^−1^ is peculiar to oxidized cytochrome *c*. [50]. Bands at ~750, 1128, and 1585 cm^−1^ strongly overlap with signals from other biomolecules (e.g., C-C skeletal stretching at 1123 cm^−1^), and they could hardly serve as unequivocal markers for cytochrome *c*. However, using a 532 nm (green) laser irradiation induces resonance Raman conditions for hemes of type *b* and *c* in mitochondrial cytochromes rather than proteins. For this reason, the band at 1313 cm^−1^ can yet be considered as mainly contributed by cytochrome *c* [51], although a contribution from proteins to its relative intensity cannot be ruled out. Accordingly, monitoring the ~750, 1313, and 1638 cm^−1^ Raman bands could give us a path to evaluating physiological states in *Candida* cells under oxidative stress. In the present study, different *Candida* species were spectroscopically screened and compared in their as-cultured state; it is thus conceivable to assume that they were free from extrinsic oxidative stress. We did not observe the oxidized heme Fe^3+^ band at 1638 cm^−1^, spectra from all investigated *Candida* species showing a silent zone in the interval 1602~1644 cm^−1^ (cf. Figure 2a–d). Note, however, that Raman spectra recorded with 532 nm laser allows obtaining signals only from reduced cytochrome *c*, its oxidized form displaying with a much less intense Raman spectra [50]. This could be the reason why the peak at 1638 cm^−1^ could not be observed. Moreover, in fixed cells, formalin may cause partial oxidation of cytochromes. Nevertheless, the presence of peaks from reduced hemes indicates the preservation of mitochondrial redox state after formalin fixation. Accordingly, differences in relative intensity for the reduced heme Fe^2+^ Raman marker at ~750 cm^−1^ should be attributed to an “intrinsic” content of reduced heme. In healthy cells, the cytochrome *c* is contained in the mitochondria and plays a key role in life functions by participating in adenosine triphosphate synthesis. In the absence of extrinsic stress, the small fractions of cytochrome *c* released by the mitochondria into the cytosol are rapidly reduced through various enzymes and reductants [52]. Variations in relative intensity of the pyrrole ring band at ~750 cm^−1^ thus represent different fractions of cytochrome *c* present in the mitochondria of different as-cultured *Candida* species. More specifically, they reflect the participation of heme Fe^2+^ molecules in non-apoptotic functions and can be taken as an intrinsic measure of the energetic state of the *Candida* cells. Note that the intensity trend for the 1313 cm^−1^ band follows only roughly the trend of the band at 750 cm^−1^ due to the possible contribution of signals from proteins. Regarding the possibility to directly resolve individual bands from DNA and RNA, the machine-learning screening of Raman sub-band components indicated only two possible choices, namely, the sub-bands at ~1194 cm^−1^ (C-CH_3_ stretching) [53] and ~1542 cm^−1^ (NH_2_ scissoring) [54]. These signals are mainly contributed (>95%) by thymidine and cytidine nucleosides, respectively (cf. labels in Figure 2). The relative intensity of these two bands, which clearly differed among different *Candida* species, could be assumed as directly determined by both DNA amount and ploidy in the studied *Candida* strains [55,56]. *Candida* species can indeed exhibit different DNA contents and encounter a variety of ploidy states [36,57]. DNA content/ploidy variations reflect distinct adaptive mechanisms in pathogenic *Candida* species [58,59]. Finally, it should be noted that fixation by formalin is known to induce DNA degradation, and a clear yield value for avoiding such damage is not available in literature. Although we have used a low fraction (4%) of formalin and quickly washed in PBS, damages to the DNA structure cannot be excluded. Therefore, no attempt was made here to extract quantitative chemical information on the DNA from the Raman spectra. 

Despite the potential importance of monitoring cytochrome *c* and DNA content of *Candida* cells by Raman spectroscopy, both these features are hardly suitable for developing a Raman procedure to identify different *Candida* species, because they are strongly influenced by cell metabolism, extrinsic stress, and fixation process. In the following, we first attempt to locate and evaluate a series of four Raman spectroscopic parameters, which directly relate to intrinsic structural features such as polysaccharides and lipids with presumably less dependence on cell stress state. Then, we quantitatively link these fingerprint parameters to the cell structure and apply them to the classification of different *Candida* species.

**Figure 3:**
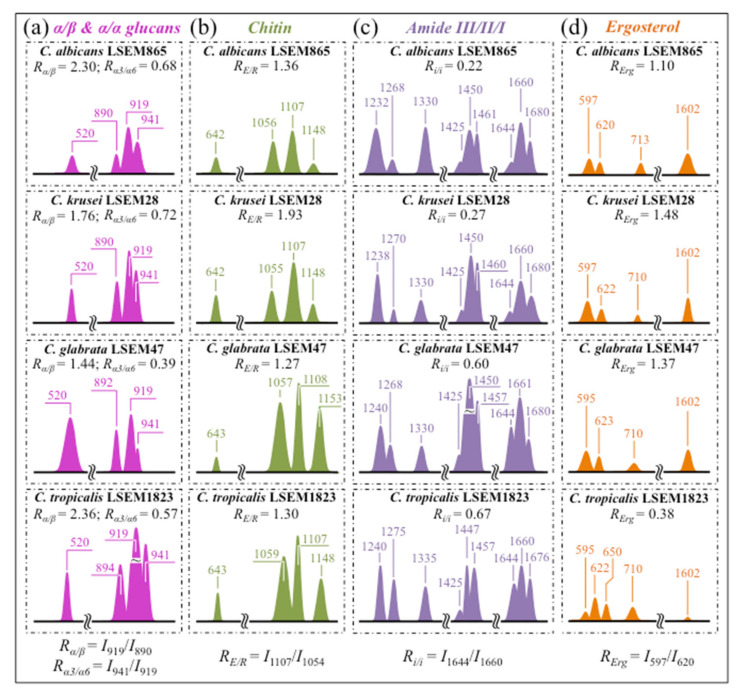
Diagrams drawn to summarize the spectroscopic differences recorded between different *C. auris* species (cf. labels); the sub-band components discussed in the text as markers of specific molecules are redrawn and compared with respect to their normalized intensities and wavenumber positions: (**a**) glucans; (**b**) chitin; (**c**) Amides I, II, and III, and (**d**) ergosterol.

**Figure 4:**
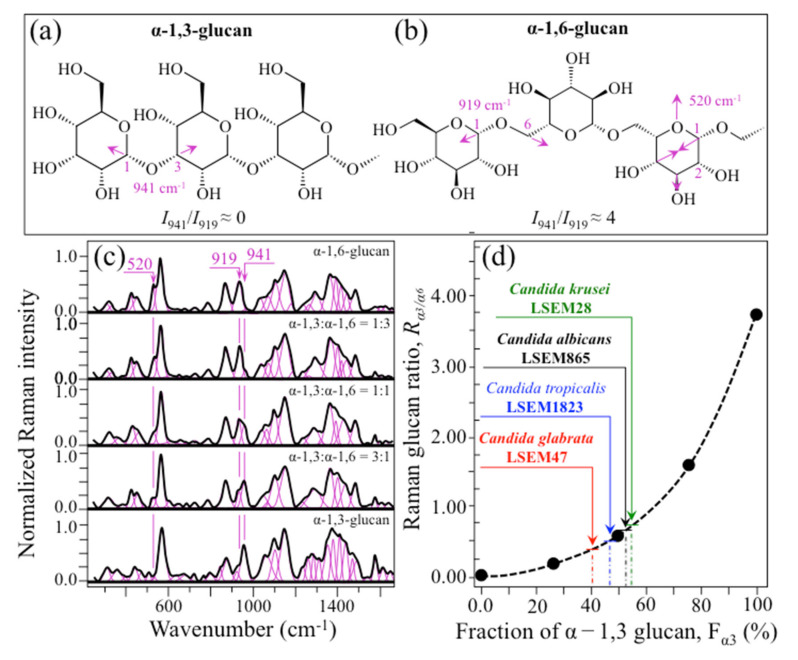
Structures of (**a**) α-1, 3-glucans and (**b**) α-1, 6-glucans with their respective vibrational fingerprints; (**c**) Raman spectra of α-1, 6- and α-1, 3-glucans and their mixtures, and (**d**) the obtained calibration plot linking the Raman intensity ratio, *R_α3/α6_* = *I*_941_/*I*_919_, to the α-1, 3-glucan fraction, *F_α3_*, from which the glucan structures of the studied *Candida* species are evaluated (cf. labels).

**Figure 5:**
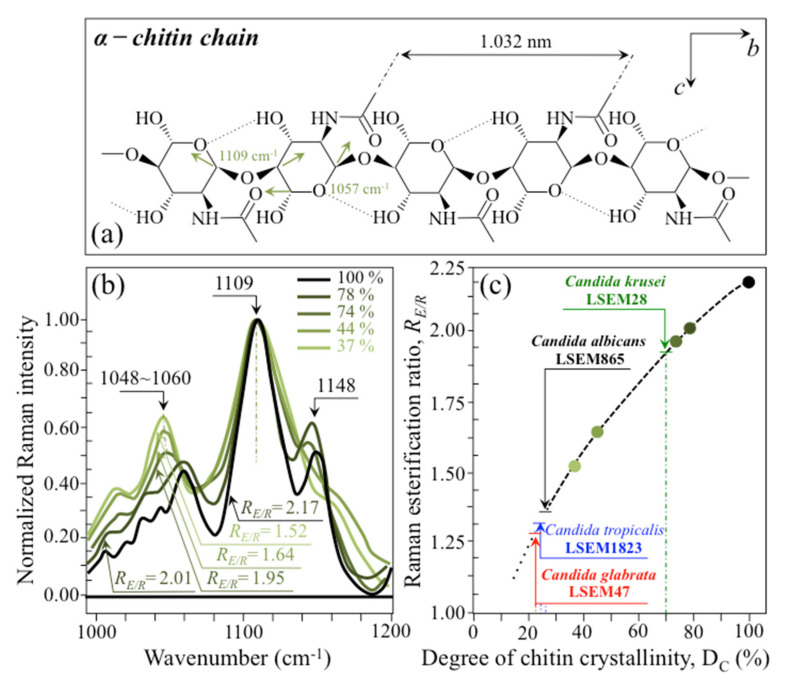
(**a**) Schematic draft of the chitin chain with its ester and ring vibrational modes; (**b**) Raman spectra of a series of chitin compounds with decreasing chain lengths in comparison with the spectrum of a reference (fully crystalline) α-chitin compound [66] (labels in inset show band wavenumbers in cm^−1^, crystallinity values by wide-angle X-ray diffraction measurements in %, and *R_E/R_* values as calculated from the Raman spectra); in (**c**), plot of the *R_E/R_* ratio as a function of percent degree of chitin crystallinity, D_C_, according to which the studied *Candida* species were evaluated (cf. labels).

**Figure 6:**
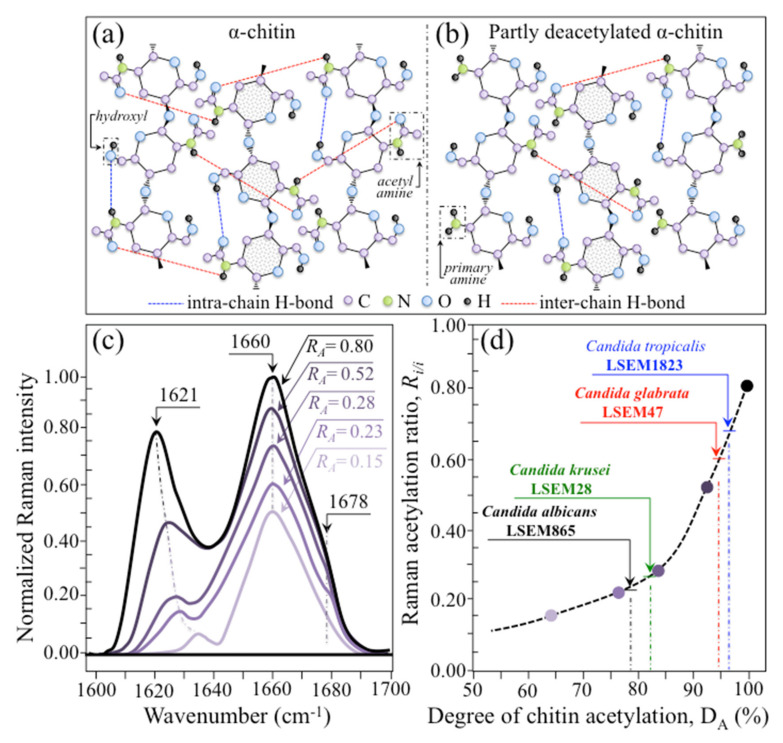
Structure of (**a**) fully acetylated and (**b**) partly deacetylated α-chitin crystals; (**c**) Amide I spectra of increasingly deacetylated chitin structures with their structure-dependent vibrational characteristics [72], which vary with decreasing the degree of chitin acetylation, D_A_ (namely, the fraction of acetyl groups present in the α-chitin structure), and (**d**) calibration curve linking the Raman acetylation ratio, *R_i/I_*, to the degree of chitin acetylation, D_A_, according to which different *Candida* species were evaluated (cf. labels).

(i) Polysaccharides—Among Raman bands of polysaccharides, fingerprints for β-1, 3-glucans and α-1, 3-glucans can be found at ~890 cm^−1^ and ~941 cm^−1^, respectively. Both bands arise from C-O-C glycosidic linkage vibrations but differ in frequency because of the different bond type and of the structural context in which the bond is embedded. The choice of the 941 cm^−1^ marker was suggested in previously published studies of α-1, 3-glucans [60,61] Accordingly, the intensity ratio of the 941 to the 890 cm^−1^ bands, *R_α/β_* = *I*_932_/*I*_890_, express the volumetric fraction of α- to β-glucans and could be taken as a structural marker for the yeast wall structure. As shown in Figure 3a, the *R_α/β_* ratio clearly differed for different *Candida* species (i.e., 2.30, 1.76, 1.44, and 2.36 for *C. albicans*, *C. krusei*, *C. glabrata*, and *C. tropicalis*, respectively). An additional spectroscopic parameter can be envisaged when considering the difference in vibrational behavior between α-1, 3-glucans and α-1, 6-glucans. Figure 4a,b shows the different structures of these two polysaccharides, respectively, and give schematic drafts of their peculiar vibrational fingerprints. As mentioned above, a vibrational signal peculiar to the α-1, 3-glucan structure is the stretching of the C1-O-C3 inter-ring bonds at 941 cm^−1^ (cf. Figure 4a). On the other hand, vibrational signals peculiar to the α–1, 6–glucan structure can be found at 919 cm^−1^ (C1-O-C6 inter-ring stretching) and 520 cm^−1^ (in-plane ring vibration) (cf. Figure 4a). We performed a Raman spectroscopic calibration by mixing α-1, 6-glucan with increasing fractions of α-1, 3-glucan, and recording the respective spectra as shown in Figure 4c. After spectral deconvolution, we computed the Raman intensity ratio, *R_α3/α6_* = *I*_941_/*I*_919_, and plotted it as a function of the α-1, 3-glucan fraction, *F_α3_*, in order to obtain a calibration curve as shown in Figure 4d. The availability of a curve linking the Raman parameter, *R_α3/α6_*, to the fraction, *F_α3_*, allows computing the fraction of α-1, 3-glucans in different *Candida* species. The percent *F_α3_* values of *C. albicans*, *C. krusei*, *C. glabrata*, and *C. tropicalis* were calculated from the respective *R_α3/α6_* values (listed in Figure 3a) as 53.7, 55.0, 40.3, and 47.2%, respectively. The ability of a specific *Candida* species to synthesize higher fractions of water-insoluble glucans (i.e., glucans rich in α-1,3- and β-1,3-glucosidic linkages) [62], as compared with water-soluble glucans (i.e., rich in α-1,6-glucosidic linkages) [63,64], is crucial in determining the structural and dynamical attributes of the yeast membrane.
(ii) Chitin backbone—The spectral region representative of C-C-C and C-O-C stretching in polysaccharides corresponds to the wavenumber interval between 950 and 1200 cm^−1^. Despite structural similarities, which reflect into similar spectroscopic characteristics, distinctions among different polysaccharides can yet be made upon using bands in this peculiar region. Chitin is structurally different from other polysaccharides because of the appearance of C-O-C bonds between neighboring rings and the presence of amide groups to partly replace OH groups as structural sub-units in the polymeric chain [65]. These structural peculiarities confer on chitin distinctive vibrational characteristics as compared to other polysaccharides. The triplet at 1054, 1107, and 1147 cm^−1^, as well as the low-frequency band at 645 cm^−1^ stand conspicuously free from overlap with other signals in the spectrum of *Candida* species, and thus could serve as fingerprints for chitin. On the other hand, as mentioned above, the band at ~1123 cm^−1^ is a cumulative one for C-C (and C-O) skeletal stretching in polysaccharides. An important detail regarding chitin-related bands lies in the distinction between signals from C-O-C groups within and between rings. The ether C-O-C stretching band between rings is seen at 1107 cm^−1^, while stretching of C-O-C bonds within rings produces a Raman signal at 1054 cm^−1^. Accordingly, the band intensity ratio, *R_E/R_* = *I*_1107_/*I*_1054_, can be taken as representative of the ratio between ether and ring C-O-C bonds in chitin. Accordingly, the *R_E/R_* ratio could be defined as “esterification ratio”. Figure 5a shows a schematic draft of the chitin chain with its ester and ring vibrational modes. Chitin structures with longer chains are expected to present a higher fraction of ether C-O-C bonds and higher crystallinity; the *R_E/R_* ratio thus reflects whether the chitin structure incorporates or not long crystalline chains. *C. krusei* appears to possess chitin structures with the highest *R_E/R_* ratio (1.93), while *C. albicans*, *C. glabrata,* and *C. tropicalis* present *R_E/R_* ratios (1.36, 1.27, and 1.30, respectively; cf. labels in inset to Figure 3b). Binias et al. [66] have systematically compared the Raman spectra of natural krill chitin with a series of chitin compounds purposely produced with decreasing chain lengths. These spectra are given in Figure 5b in comparison with the spectrum of a reference (fully crystalline) α-chitin compound (INTIB GmbH, Freiberg, Germany) [67] after normalization to the ether C-O-C stretching signal, seen at the constant wavenumber of 1109 cm^−1^. The *R_E/R_* ratio clearly tended to decrease with decreasing crystallinity (cf. crystallinity values by wide-angle X-ray diffraction measurements in inset to Figure 5b). Note also that unlike the ester C-O-C signal, whose wavenumber remained nearly constant for different degrees of crystallinity, the ring C-O-C signal underwent a clear shift of several wavenumbers (1048~1060 cm^−1^) with decreasing crystallinity. A plot of the *R_E/R_* ratio as a function of percent degree of chitin crystallinity, D_C_, is drawn in Figure 5c, based on the spectra shown in Figure 5b [66,67,68]. According to this parameter, the chitin structure of *C. krusei* met the crystalline value closest to standard α-chitin (~70.0%), while *C. albicans*, *C. tropicalis*, and *C. glabrata* presented conspicuously lower D_C_ values (25.8, 24.6, and 22.4%, respectively; cf. Figure 5c). The length of a chitin dimer is 1.023 nm [68] and the average length of a standard α-chitin microfibril is ~33 nm; a chain of fully crystalline α-chitin thus containing on average 64 residues [69]. Assuming the average chain length as proportional to the crystallinity value, it is concluded that, with the exception of *C. krusei* (average chain length estimated as ~45 residues), *C. albicans*, *C. glabrata*, and *C. tropicalis* incorporate only a small subgroup of crystalline α-chitin allomorph to form rigid microfibrils; whereas the remainders take a different allomorphic structure or just lie in a considerably disordered state due to either unfavorable conformations or unstable hydrogen-bonding patterns [70].
(iii) Chitin sub-units—Amides I, II, and III signals, found in the wavenumber intervals 1600~1700 cm^−1^, 1400~1500 cm^−1^, and at around 1300 cm^−1^, respectively, are peculiar to the N-acetyl group of chitin (cf. labels in Figure 1). The intensities of these signals are thus related to the level of acetylation/deacetylation of the membrane structure of different *Candida* species. The occurrence of a doublet for the Amide I, which we see at ~1644 and 1660 cm^−1^ in all species (cf. Figure 3c), has been attributed to the existence of two different types of hydrogen bonds in α-chitin crystals: intermolecular (C=O…HN) and intramolecular (C=O…HO(C6) or C=O…HN) hydrogen bonds (cf. schematic draft in Figure 6a) [71,72,73]. This doublet gives an opportunity to classify different *Candida* species according to their degree of acetylation. Additional bands in the Amide I region, which are seen as shoulders at 1675~1680 and >1688 cm^−1^, are assigned to C=O stretching and associated with the presence of different chitin secondary structures or defects in small crystalline chitin fibrils, respectively (cf. Figure 2) [71]. The degree of acetylation, namely, the fraction of acetyl groups present in the α-chitin structure, is linked to the crystalline structure of chitin: a high degree of acetylation favoring the stable α-chitin crystalline structure through a network of hydrogen bonds that the amine acetyl groups can form with NH groups (Figure 6a) [74,75,76]. Conversely, chitins with low degrees of acetylation and, thus, with higher probability of primary amine groups in their structure are more likely to take different allomorphs or more defective structures (cf. Figure 6b). Following Zhang et al. [72], α-chitin structures can be characterized with respect to their degree of acetylation by means of Raman spectroscopy. The Amide I spectra of increasingly deacetylated chitin structures (i.e., containing decreasing fractions of acetyl groups) are shown in Figure 6c. This spectral zone shows three main structure-dependent vibrational characteristics, which vary with decreasing the degree of chitin acetylation, D_A_: [72] (i) a low-frequency band, seen at ~1621 cm^−1^ in fully acetylated α-chitin structure, with a decreasing relative intensity and progressively shifting toward higher wavenumbers; (ii) a band located at ~1660 cm^−1^, decreasing in relative intensity but keeping its spectral location unchanged; and, (iii) a shoulder at 1678 cm^−1^, showing a decreasing relative intensity. In the Raman spectra shown in Figure 2, the spectral zone between 1602 and 1644 cm^−1^ did not show Raman signals in any of the studied *Candida* species; a band seen at 1644 cm^−1^ was thus assumed as the low-frequency Amide I signal. As mentioned above, one could take the relative intensity ratio, *R_i/i_* = *I*_1644_/*I*_1660_, referred to as the Raman ratio of intermolecular to intramolecular hydrogen bonds, as a parameter measuring the degree of acetylation of α-chitin. Data from reference chitin structures with known structural characteristics [72] allow building a calibration curve according to which the *R_i/i_* values measured in the Raman spectra of different *Candida* species can be evaluated and compared (Figure 6d). This procedure reveals that *C. tropicalis* and *C. glabrata* possess a relatively high degree of acetylation in their α-chitin structures (i.e., D_A_ = 95~97%), while *C. krusei* and *C. albicans* are characterized by relatively more deacetylated α-chitin structures (i.e., D_A_ = 78~82%). Although a high degree of acetylation favors the formation of α-chitin crystalline structure, *C. krusei*, which showed by far the highest degree of α-chitin crystallization (cf. D_C_ = 70%; cf. Figure 5c) among the studied *Candida* species, was only the second lowest in degree of acetylation (D_A_ = 82%; cf. Figure 6d); vice versa, *C. tropicalis* and *C. glabrata*, both low in D_C_, were the two highest species in D_A_. This means that the low D_C_ values of the latter two species are rather due to the presence of different chitin allomorphs than to a disordered state of the α-chitin allomorph itself. As previously mentioned, the structure of α-chitin is stabilized simultaneously by intra- and inter-chain hydrogen bonding (cf. Figure 6a) [71,72,73,77,78]. However, while the former type of hydrogen bond is present in all chitin allomorphs, the latter one is relatively rare in the γ-chitin and completely absent in β-chitin [77,79,80]. Accordingly, *C. krusei* contains high-crystallinity α-chitin in its most stable, ordered, and tightly packed structure, but it might also incorporate a fraction of β-chitin with no inter-chain bonds, which leads to the relatively low D_A_ measured by Raman spectroscopy. Kaya et al. [81] have measured and compared the Raman spectra of α-, β-, and γ-chitin pure allomorphs. An examination of these spectra revealed that, even on pure compounds, very few spectral details could be available for differentiating different allomorphs from Raman analyses. The machine-learning procedure adopted in this paper located only one relatively hidden but yet resolvable band at ~694 cm^−1^ that could unequivocally represent the β-chitin allomorph (located with a green asterisk in Figure 2). The relative intensity of this band, which arises from ring deformation [65], is indeed stronger in the spectrum of *C. krusei* than in different *Candida* species.
(iv) Ergosterol—Regarding lipids, the machine-learning algorithm located two low-frequency bands (at 594 and 620 cm^−1^; both related to in-plane ring deformation modes) as mainly (>95%) contributed by steroid ergosterol [82]. Of the two main C=C stretching bands at 1602 cm^−1^ (within rings) and 1666 cm^−1^ (in acyl chains), only the former could be detected as a clear shoulder, while the latter undergoes strong overlap with Amide I signals. The relatively intense and isolate band at ~713 cm^−1^ is contributed by ergosterol (cumulative of C-H bending and ring deformation) [82], but it also contains non-negligible contributions from D-arabitol, chitin, and adenine. Judging from the cumulative (relative) intensities of the 594/620 cm^−1^ doublet in the spectra of different *Candida* species (cf. Figure 3d), the fraction of ergosterol appeared to be comparable in all species. However, the trend of ergosterol bands showed important differences in *C. tropicalis*, as compared with other *Candida* species:
  (a) The doublet at 597/620 cm^−1^ showed an inversion in relative intensity as compared to the other three *Candida* species investigated, with the low frequency band becoming more intense than the higher frequency one.
  (b) A new band, seen as a shoulder band, was clearly detected at ~650 cm^−1^, which was not seen in any spectrum of the other *Candida* species (cf. red asterisk in Figure 2d).
  (c) The shoulder band at 1602 cm^−1^, which was clearly detected in *C. albicans*, *C. krusei*, and *C. glabrata*, conspicuously disappeared in *C. tropicalis*.

**Figure 7:**
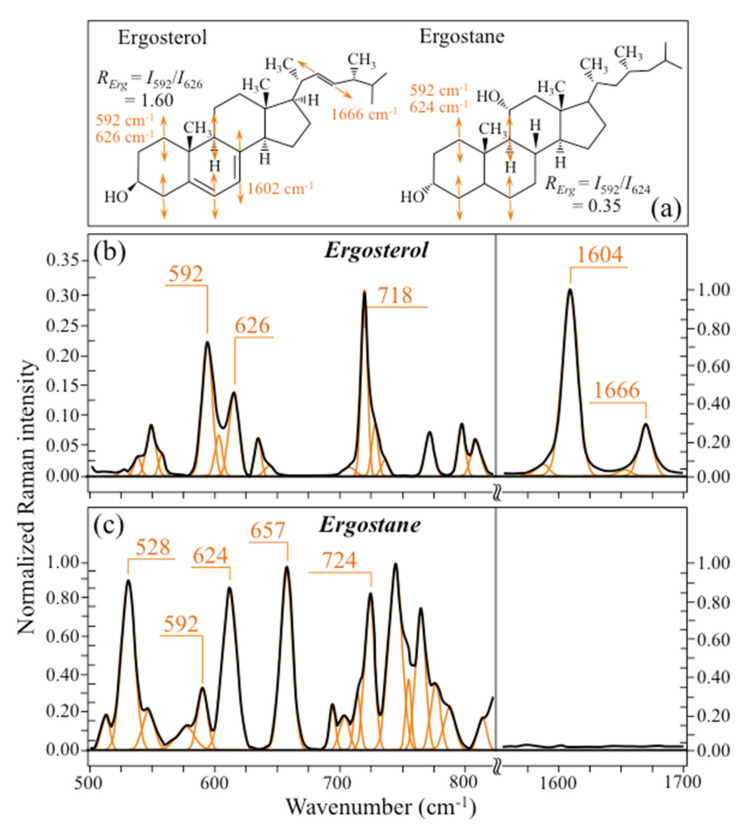
(**a**) Structures of ergosterol and ergostane molecules with their characteristic vibrational fingerprints and their respective Raman spectra (in (**b**) and (**c**), respectively).

These three spectroscopic features suggest the prevalence of an ergostane structure in C. *tropicalis*. As a matter of fact, the ergostane molecule, whose structure and Raman spectrum are compared to those of ergosterol in Figure 7 [83], lacks -C=C- double bonds in both the second sterol ring and the acyl chain (cf. structures compared in Figure 7a). The spectroscopic consequences of this structural difference are indeed: (a) an alteration of ring deformation bands at low frequency, leading to the observed inversion in relative intensity of the 597/620 cm^−1^ doublet; (b) the appearance of a new ring deformation band at 650 cm^−1^, and (c) a complete lack of the -C=C- stretching bands both in ring and alkyl-side chain at 1604 and 1666 cm^−1^, respectively (cf. Figure 7b,c for ergosterol and ergostane, respectively). Note that, as mentioned above, it is not possible to unequivocally monitor variations in the 1666 cm^−1^ and other main ergostane bands in the *Candida* spectra due to overlap with predominant vibrational signals from other molecules. One possible parameter for classifying the ergosterol structure of different *Candida* species is the ratio between the relative intensities of the 597/620 cm^−1^ doublet, *R_Erg_* = *I*_597_/*I*_620_. *R_Erg_* values >1 should confirm a lipid structure with a preponderant ergosterol fraction, as in the case of *C. krusei*, *C. glabrata*, and *C. albicans* in decreasing order (cf. *R_E_* values in inset to Figure 3d). Conversely, *R_Erg_* values <1 should mark an increasing predominance of the ergostane structure, as in the case of *C. tropicalis*. *R_Erg_* ratios can be converted into values of (percent) fraction of ergostane, *F_Es_*, by assuming a linear relationship between *R_Erg_* ratios as measured on ergosterol and ergostane pure compounds (i.e., 1.60 and 0.35, respectively; cf. Figure 7b,c). The assumption of a linear relationship between the two values implies that the ring deformation bands in ergosterol and ergostane experience the same Raman cross-section. This is considered to be the case here because these two different structures give signals at the same wavenumbers, although they display different relative intensities.

**Figure 8:**
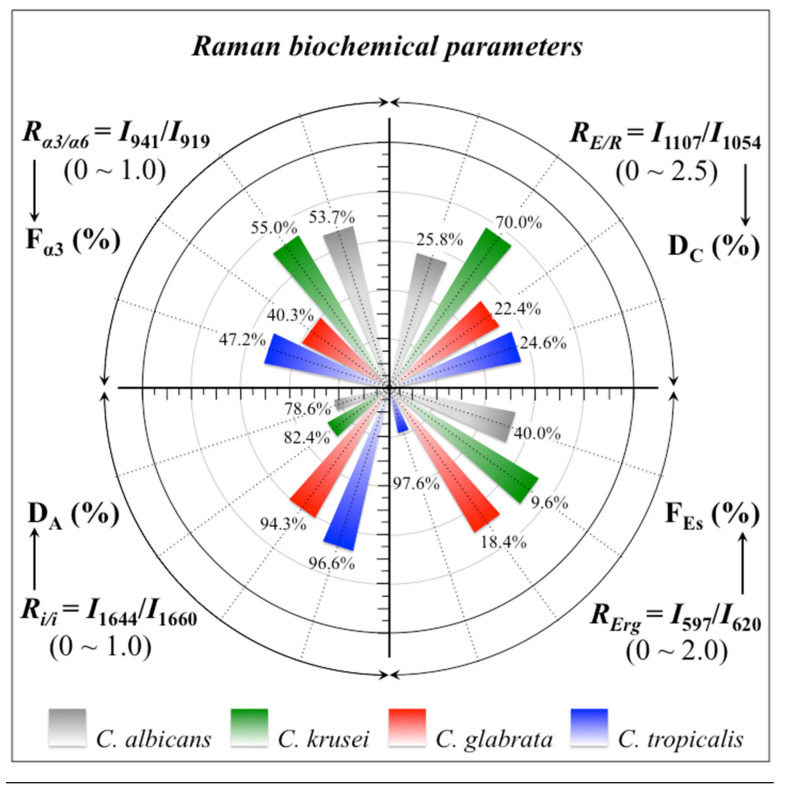
Polar plot of four selected Raman biochemical parameters for the studies *Candida* species: *R_α3/α6_* = *I*_941_/*I*_919_, *R_E/R_* = *I*_1107_/*I*_1054_, *R_i/i_* = *I*_1644_/*I*_1660_, and *R_Erg_* = *I*_597_/*I*_620_, quantitatively linked to the fraction of α–1, 3–glucans, F_α3_, the degree of chitin crystallinity, D_C_, the degree of chitin acetylation, D_A_, and the fraction of ergostane, F_Es_, respectively (cf. labels in inset).

In summary, Raman spectroscopic analyses located a series of four spectroscopic parameters, which we shall refer to as Raman biochemical parameters, representing vibrational fingerprints of polysaccharides and lipids for different *Candida* yeast species. These four Raman biochemical parameters, namely, *R_α3/α6_* = *I*_941_/*I*_919_, *R_E/R_* = *I*_1107_/*I*_1054_, *R_i/i_* = *I*_1644_/*I*_1660_, and *R_Erg_* = *I*_597_/*I*_620_, have been quantitatively linked to the fraction of α-1, 3-glucans, *F_α3_*, the degree of chitin crystallinity, D_C_, the degree of chitin acetylation, D_A_, and the fraction of ergostane, *F_Es_*, respectively. All these parameters are summarized in Figure 8 as a polar plot including the four oral *Candida* species investigated in this study. As seen, the selected parameters vividly reflect a clear diversification in key structural characteristics, which foresees a breadth in functional diversity among different *Candida* species.

### 2.3. Raman Imaging of Oral Candida Species

**Figure 9:**
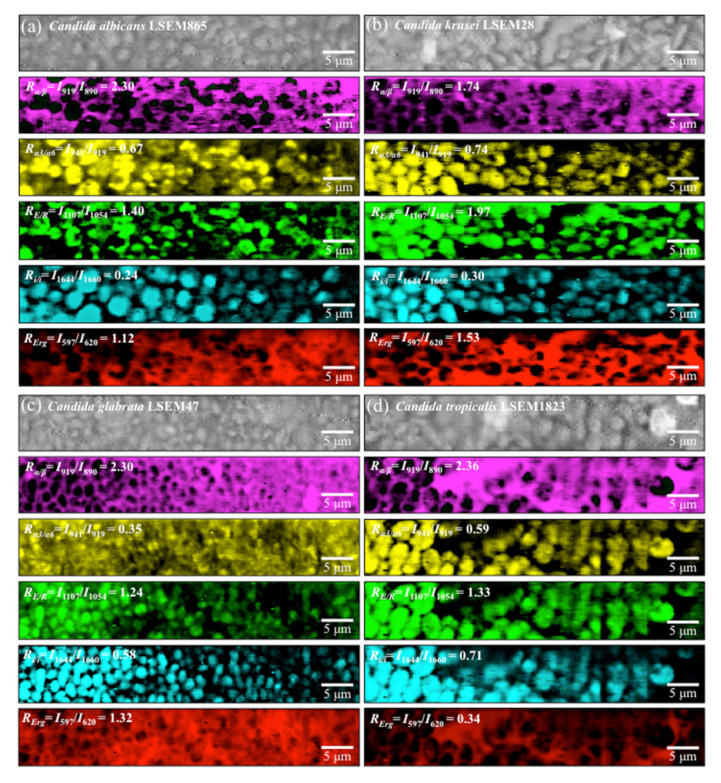
Raman maps of the five selected parameters *R_α/β_* = *I*_919_/*I*_890_, *R*_α3*/*α6_ = *I*_941_/*I*_919_, *R_E/R_* = *I*_1107_/*I*_1054_, *R_i/I_* = *I*_1644_/*I*_1660_, and *R_Erg_* = *I*_597_/*I*_620_ for (**a**) *C. albicans*, (**b**) *C. krusei*, (**c**) *C. glabrata*, and (**d**) *C. tropicalis*.

Raman mapping was performed with micrometric spatial resolution in order to image the four Raman biochemical parameters described in the previous section. This additional effort was made in the attempt to validate the findings obtained by analyzing the average spectra shown in Figure 2. Raman maps of the five parametric ratios, *R_α/β_* = *I*_919_/*I*_890_, *R_α3/α6_* = *I*_941_/*I*_919_, *R_E/R_* = *I*_1107_/*I*_1054_, *R_i/i_* = *I*_1644_/*I*_1660_, and *R_Erg_* = *I*_597_/*I*_620_ are shown in Figure 9a–d for *C. albicans*, *C. krusei*, *C. glabrata*, and *C. tropicalis*, respectively. The total number of Raman spectra collected in several maps collected on each *Candida* species was in the order of 106 over a total area of ~103 μm^2^. The “mapping approach” is complementary to the “average approach” followed in the previous section, in which representative spectra were obtained upon averaging over tens of spectra collected with a relatively low spatial resolution (20× optical lens) to cover relatively large areas of the clade cultures (in the order of mm^2^) with high spectral resolution. Average values for the four selected Raman biochemical parameters are given in an inset to each section of Figure 9. In addition to revealing details of the spatial distribution of individual molecules, a remarkable result was that the two complementary “mapping” and “average” approaches gave results consistent to each other with differences <10%. This result can be interpreted as a confirmation of the reliability of the Raman method and its suitability to systematically analyze different *Candida* species from the oral cavity and decode their structural differences at the molecular scale.

### 2.4. Chemometric Results and Barcode Construction

**Figure 10:**
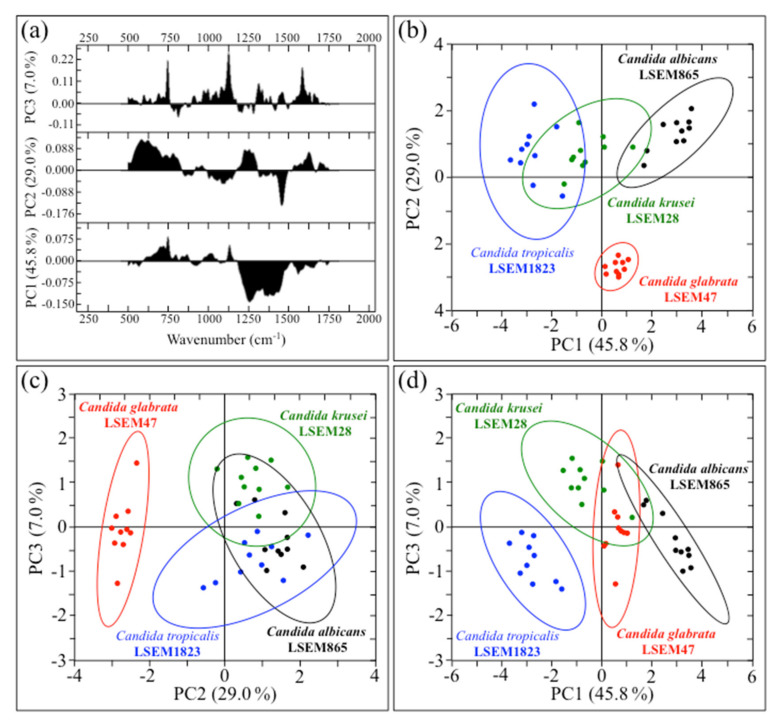
(**a**) First, second, and third principal components (PC1, PC2, and PC3, respectively) of PCA analysis performed on Raman spectra recorded on the studies *Candida* species; in (**b**–**d**), three different combinations of loading vectors are displayed, which refer to the spectral region 300~1800 cm^−1^.

For each *Candida* species investigated, we selected ten locations ~20 μm^2^ in size, each containing 400 spectra, which were then averaged to obtain a single spectrum for each location. Ten spectra (each average of 400) per each species were then subjected to PCA analyses. Figure 10a shows the first, second, and third principal components (PC1, PC2, and PC3, respectively) of PCA analyses performed on Raman spectra recorded according to the above-described procedure. In Figure 10b–d, three different combinations of loading vectors are displayed, which refer to the entire spectral region 300~1800 cm^−1^. As seen in these plots, PCA analyses show that *C. glabrata* and *C. tropicalis* data sets can be separated by appropriately selecting loading vectors. However, the PCA analysis also points out that it is not possible to distinguish between *C. albicans* and *C. krusei* using Raman spectroscopy. The present application of the PCA statistical method, which reduces the dimensionality of the Raman data matrix to two orthogonal variables, gives clear evidence of the limitations that standard chemometric analyses face in fully identifying oral *Candida* species upon discriminating among their Raman spectra. These limitations call for an alternative approach for oral *Candida* speciation.

**Figure 11:**
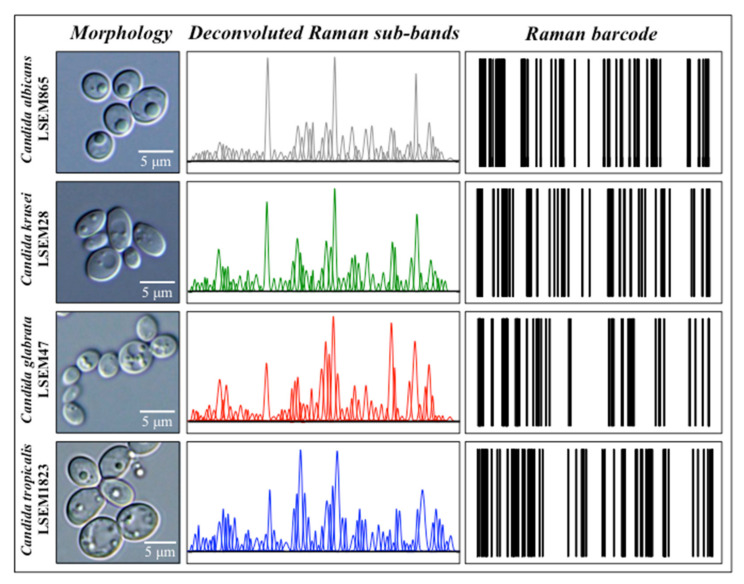
High-resolution DIC micrographs, series of deconvoluted Raman sub-bands for the four studied *Candida* species, and their conversion into Raman barcodes according to the algorithm described in Section 4.5 (cf. labels in inset).

Raman barcoding is presented here as an alternative approach to chemometric PCA analysis in identifying oral *Candida* species. This approach was applied to averages obtained from a series of 20 Raman spectra collected with a 20× optical lens with high spectral resolution on each of the four species of oral *Candida* investigated. The Raman barcode was created in the attempt to obtain a greater depth in capturing structural details than mere spectral morphology. This approach relies on the collection of a relatively lower number (i.e., 20 rather than 400) of spectra collected over larger (i.e., 20× rather than 100×) areas of the sample with high spectral resolution. After averaging the collected spectra to obtain a reliable (average) spectrum per each *Candida* species, a machine-learning-based spectral deconvolution is applied to obtain a sequence of Lorentzian–Gaussian sub-bands for each species under scrutiny. Then, a Raman barcode is attached to each sub-band sequence according to the algorithm described in Section 4.5. Accordingly, the Raman barcode contains subtle details of the molecular structure that could promptly become available by barcode scanner apps. Figure 11 shows a series of deconvoluted Raman sub-bands for the four studied *Candida* species and their conversion into Raman barcodes according to the algorithm described in Section 4.5. The series of Raman sub-bands in Figure 11 are the same as those shown in the deconvoluted spectra of Figure 2. As seen, the constructed Raman barcodes amplify subtle spectral differences, which become translated into line patterns clearly distinguishable even by the naked eye.

## 3. Discussion

### 3.1. Interpretation of Raman-Decrypted Molecular-Scale Information

The Raman analyses of oral *Candida* yeasts presented in this paper have unveiled clear differences in structure for carbohydrate polymers, namely, glucans and chitin that compose the yeast membrane. These molecules are known to harmonize with each other to maintain integrity and physical strength of the cell wall, with their assembly being essential in the capacity of each *Candida* species to manipulate the cell walls in dynamic response to external stress [84]. This study spectroscopically monitored the carbohydrate structure of different *Candida* species in absence of stress. In addition to allowing yeast speciation, these observations could help clarifying how different *Candida* species are structured to resist stress, thus laying the basis for monitoring stress-induced variations in future studies using the same Raman approach.

Glucans are the major yeast cell-wall components with a predominance of linear β-1, 3-linked long chains and a minor fraction (<10%) of β-1, 6-linked side branches [82]. A recent magic-angle spinning solid-state NMR by Kang et al. [70] has revealed the presence (and emphasized the importance) of a fraction of α-1, 3-glucans on otherwise α-1, 6-linked mannose units, which can be found in the backbone structure of mannan. Mannan is a highly branched polysaccharide linked to proteins and a major component of the outmost layer of cell walls [85,86]. Using the notions on the supramolecular assembly of the yeast cell walls given in published studies [69,70,84,85,86,87,88], the present Raman spectroscopic assessments enable to discuss the links between the fractions of these complex biomolecules to cell walls’ properties. 

Key structural/functional characteristics of yeast cell walls, as reported by other authors [69,70,87,88] can be summarized in the following six points:

(i) The α-1, 3-glucan is by far the most rigid molecule in the cell-wall structure (followed by chitins and β-glucans), while linear β-1, 3-linked long chains possess the highest level of hydration and mobility [70].
(ii) Chitin and α-1, 3-glucan intertwine to form a rigid and hydrophobic scaffold surrounded by a matrix of pliable and hydrated matrix of β-glucans [70].
(iii) The cell-wall surface is coated with a highly dynamic shell consisting of glycoproteins and a minor fraction of β-glucans [70].
(iv) Non-microfibrillar chitin and β-1, 6-glucan soft chains might link to rigid chitin microfibrils with sufficient lengths to ensure cell-wall flexibility [69].
(v) Different chitin allomorphs coexist as tightly packed chains at the sun-nanometer scale rather than existing in separated longitudinal domains [70].
(vi) As a key characteristic of the polymorphic structures and of the heterogeneous dynamics of cell-wall molecules, the presence of a fraction of α-1, 3-glucans present in the outmost layer of cell walls has the function to block immune recognition of β-glucan receptors in the host cells [87,88].

Following the above notions and according to our Raman approach, one could take the product of the two parameters *R_α/β_* × *R_α3/α6_* as a measure of efficiency in the bi-functionality of α-1, 3-glucans, namely, walls’ rigidity/hydrophobicity and immune recognition blocking (cf. above points (i), (ii), and (vi)). The obtained order among the investigated *Candida* species from the richest in α–1, 3–glucan to the poorest is: *C. albicans*, *C. tropicalis*, *C. krusei*, and *C. glabrata* (with products *R_α/β_* × *R_α3/α6_* equal to 1.54, 1.34, 1.26, and 0.56, respectively). Remarkably, *C. glabrata*, i.e., the lowest in ranking for α-1, 3-glucan content, shows also the lowest chitin chain length (*R_E/R_* = 1.27) and the second highest degree of acetylation (*R_i/i_* = 0.27). This set of unique cell-wall structural characteristics confers to this species the highest cell wall flexibility to be exploited under stress conditions to compensate for low hydrophobicity. *C. glabrata* is the second most common fungal pathogen isolated from humans [20,89], and has developed robust resistance to a range of environmental stresses, which include cationic/osmotic, oxidative, and nitrosative stresses [90]. The present Raman data, in agreement with and complementary to genomic analyses [91], provides an interpretation of the structural reasons behind the development of a strong environmental stress resistance. Conversely, *C. krusei*, with its relatively high fraction of α-1, 3-glucan (*R_α/β_* × *R_α3/α6_* = 1.26) and the longest (*R_E/R_* = 1.27) and poorly acetylated chitin chains (*R_i/i_* = 0.27) among oral *Candida* species, appears to be, structurally, the least equipped for a flexible structural response of its cell walls. The osmotolerance of this species is indeed mainly due to its superior capacity to synthesize glycerol as a compatible substance to balance the extracellular hyperosmotic stress [92,93].

### 3.2. Limits of Chemometrics and Leverage of the Barcode Approach

PCA-based chemometrics is a widely used tool in biomedical spectroscopy, which enables to assess the statistical validity of the obtained results [94,95,96]. However, when applied as a purely empirical correlation based on the mere morphology of the acquired spectra, this approach might show strict limits in its applicability. In this study, we applied the PCA method to 10 Raman spectra obtained as averages of sets of 400, which were retrieved from Raman maps collected on each investigated *Candida* species. The PCA method failed in the speciation of two over four *Candida* species under examination.

As an alternative approach, we proposed a Raman barcode procedure, which used averages of 20 high spectrally resolved Raman spectra collected with lower spatial resolution. The Raman barcode method, which successfully speciated all studied *Candida* species, entails statistical reliability from a physically sound band deconvolution. As explained in Section 4.4, we used an automatic solver that selects compounds from a library of spectra from basic compounds and fits their sum to the average experimental spectrum with preserving relative intensities, spectral positions, and full-width at half-maximum values for the individual sub-bands of each elementary compound. 

Barcodes are already used in medical procedures to reduce the risk of human error during medication administration (e.g., overdosing patients or missed medication errors) [97]. Their use to classify pathogenic species has already been applied to DNA barcoding of bacterial strains and yeast isolates [98,99,100,101]. DNA barcoding, which nowadays represents a global initiative for species identification, is based on sequencing short DNA sequence markers at selected loci. Compared to DNA barcoding, the main advantage of Raman barcoding is the possibility of a much faster identification, which could enable a nearly real-time on-site identification of the pathogenic species, which is not possible with DNA analyses. While this study clearly shows the feasibility of the Raman barcoding approach for the most widespread *Candida* species of the oral cavity, the implementation of this technology into the clinical practice necessarily requires the development of relatively inexpensive small-size Raman devices, capable to generate Raman barcodes with sufficient spectral resolution. This development is certainly possible, but such specialized device is not yet available. At the present time, the present authors are collecting representative Raman spectra and building a library of Raman barcodes for all the available *Candida* clades and isolates. However, the need for infrastructural investments in Raman devices reduced in size and upgraded in efficiency could be seen as the main present limitation in extrapolating the proposed Raman barcoding approach to clinical setting.

### 3.3. Clinical Implications of the Proposed Raman Barcode Technology

The main advance of the proposed Raman technology is represented by the possibility of classifying in real-time and on-site *Candida* populations sampled from individual patients in the immediate follow-up of an outpatient procedure. This possibility could support the clinicians in choosing the most effective drug to be used for specifically suppressing invasive *Candida* infections and for minimizing the development/spreading of drug resistant isolates. In addition, the Raman barcode procedure is also capable to make promptly available deeper information about the structural characteristics of the specific isolates under scrutiny. This information could be particularly important when classifying clinical samples, which could differ (beyond genomics) due to the specific environmental conditions of the patient’s body under which they developed. The availability of deeper knowledge on the specific infections could allow pharmaceutical industries to develop new drugs, clinicians to translate results into meaningful implications for the treatments, and patients to be addressed with a more detailed dialogue-based approach upon communicating the test results in a factual way, best supported by scientific data. It should also be noted that the presented technology, once properly developed, could have relevance beyond oral candidiasis. In support of this assertion, one could notice that *C. krusei*, which is also studied here, has been found capable to induce severe levels of candidemia after developing echinocandin resistance during caspofungin therapy [102]. Moreover, Raman analyses similar to those proposed in this study have previously been applied to *C. auris* clades II and III in comparison with *C. albicans*, and showed capable to reveal structural details at the molecular scale, which relate to both virulence and drug resistance characteristics [32,33].

Finally, the authors are aware that integration of a new risk assessment technology into clinical practice is usually accompanied by several challenges; one is certainly represented by the need to integrate the Raman technology into the existing routines, which in turn requires physicians (and patients as well) to understand the new technology in relation to their daily practice.

## 4. Materials and Methods

### 4.1. Candida Species

*Candida albicans* LSEM865 (*C. albicans*), *Candida krusei* LSEM28 (*C. krusei*), *Candida glabrata* LSEM47 (*C. glabrata*), and *Candida tropicalis* LSEM1823 (*C. tropicalis*) cells were provided by Teikyo University. *Candida* species were cultured in brain heart infusion (BHI) broth (NISSUI PHARMACEUTICAL Co., Ltd., Tokyo, Japan) at 36 °C for 24 h on round glass bottom dishes 35 mm in diameter (MatTek Life Sciences, Ashland, MA, USA). All *Candida* species were in their subconfluent status at the time of their spectroscopic characterization. The yeast cells were fixed with 4% formalin and quickly washed in phosphate buffered salts (PBS). 

### 4.2. Differential Interference Contrast (DIC) Microscopy

*Candida* species were cultured in potato dextrose agar at 36 °C for 48 h. A sterile inoculation loop was used to pick a colony of yeast up, and yeast cells were mounted in distilled water on a microscope slide (Matsunami Glass Ind., Ltd., Kishiwada, Osaka, Japan). The cells were then observed with a DIC microscopy (BX53, OLYMPUS Co., Tokyo, Japan) at magnification of 400~1000×.

### 4.3. In Situ Raman Spectroscopy

Raman spectra were collected in situ on yeast cells of all species. Spectra were obtained using a dedicated instrument (LabRAM HR800, Horiba/Jobin-Yvon, Kyoto, Japan) operating with a 20× optical lens with the spectroscope set in confocal mode. The spectroscope was equipped with a holographic notch filter enabling efficient and high spectrally resolved acquisitions. Excitation was made with a 532 nm solid-state laser source operating at 10 mW. A spectral resolution of better than 1 cm^−1^ was achieved by using an internal reference (neon emission) to calibrate the spectrometer. The Raman scattered light was monitored by a single monochromator connected with an air-cooled charge-coupled device (CCD) detector (Andor DV420-OE322; 1024 × 256 pixels). The acquisition time for a single spectrum was typically 10 s. Twenty spectra were collected at different locations on a round sample plate 35 mm in diameter over a total area of ~2 mm^2^ for each *Candida* species, and averaged in order to obtain a representative spectrum for each species.

Reference Raman spectra, collected on pure compounds, were stored in a library. The reference library compilation contained more than 40 elementary compounds (simply referred to as the “library”, henceforth), including polysaccharides (e.g., chitin, β-1,3-glucans, β-1,6-glucans, α–1, 3–glucans), mono- and disaccharides (e.g., trehalose, β-D-glucose, D-dextrose), lipids (e.g., triolein, trilinolein, 1,2-dipalmitoyl-L-α-lecithin), polyols (e.g., D-(+)-Arabitol and L-(-)-Arabitol), and other key molecules such as adenine, ergosterol, and glycine. Reference α-1,3-glucan samples were obtained from Tokyo University [40]. The spectra from the pure compounds were collected with a highly resolved spectrometer (T-64000; Jobin-Ivon/Horiba Group, Kyoto, Japan) equipped with a nitrogen-cooled charge-coupled device detector (CCD-3500V, Jobin-Ivon/Horiba Group, Kyoto, Japan). The excitation source in these latter experiments was a 514 nm line of an Ar-ion laser operating with a nominal power of 200 mW. The spectral resolution was better than 1 cm^−1^.

Raman imaging for all *Candida* species was obtained using a dedicated Raman device (RAMANtouch, Nanophoton Co., Minoo, Osaka, Japan) operated in microscopic measurement mode with confocal imaging capability in two dimensions. This Raman microscope can achieve ultra-fast simultaneous image acquisition of up to 400 spectra. The spectroscope was specially designed to be compatible with cells’ life. It used an excitation source of 532 nm. The spectral resolution was ~2 cm^−1^ (spectral pixel resolution equal to 0.3 cm^−1^/pixel) with an accuracy in laser spot location of 100 nm. Raman hyperspectral images were generated using commercially available software (Raman Viewer, Nanophoton Co., Minoo, Osaka, Japan). With the purpose of avoiding possible distortions, the Raman images were generated using intensity ratios in the normalized spectra. In order to minimize errors related to spectral resolution and possible shifts in band position, we used the average intensity of the pixels at the band nominal location ±3 pixels, rather than single pixel intensity. Maps were generated on selected areas of the samples with adopting a lateral displacement of 500 nm for the laser focal point.

### 4.4. Machine-Learning Algorithm for Spectral Deconvolution

The experimentally obtained Raman spectra were preliminary treated with a baseline subtraction procedure and then automatically deconvoluted into a series of Gaussian–Lorentzian sub-bands. Baseline subtraction and deconvolution procedure were performed using options available in commercial software (LabSpec 4.02, Horiba/Jobin-Yvon, Kyoto, Japan) by following exactly the same criteria for all spectra collected on different species. The software applied a polynomial-fitting criterion for baseline subtraction. All spectra were analyzed for their relative intensity after normalization to the glucose ring signal (seen at 478 cm^−1^). Deconvolution into sub-bands was described in detail in previous works [32,35]. Briefly, average spectra were fitted by means of an automatic solver, which exploited a linear polynomial expression of Lorentzian–Gaussian functions and adopted a working algorithm to match the experimental spectrum for minimum scatter. A computer program was built up for selecting a series of deconvoluted sub-bands from pre-selected compounds belonging to the library, including mono-, di-, and polysaccharides, specific lipids, polyols, and other key molecules, pre-selected according to previously published literature on the structure of *Candida* species. Upon obeying a pre-selection of the component molecules from the library, the algorithm located the best fit to the experimental spectra. The computational procedure preserved relative intensities, spectral positions, and full-width at half-maximum values for individual sub-bands of the deconvoluted spectra from each elementary compound (i.e., within ±3 cm^−1^, considering the resolution of the spectrometer and the possibility of slight alterations in molecular structure). The adopted criteria for the selection of band positions, relative intensity, and bandwidths provided a number of mathematical constraints that allow a univocal deconvolution of the experimental spectra. In the case where specific sub-bands were located, which the solver could not fit according to the pre-selected library, a new compound was selected from the library to match the unknown band following the same criteria as described above and by newly adjusting for the overall intensity contribution of each elementary compound. Within the given constraints, the software located a best fit for the experimental spectrum.

Regarding the details of the machine-learning algorithm and related training/validation procedures, we used a K-means clustering algorithm based on sets of 4 × 10^4^ spectra obtained from Raman imaging using arrays of 100 arrays of 400 spectra. On all investigated samples, the algorithm was able to identify the main cluster related to the presence of different *Candida* species.

### 4.5. Chemometric Analysis and Barcoding

Statistical analyses and visualization of the large Raman data sets obtained during high-resolution imaging were performed according to principal component analysis (PCA). The PCA analysis, which was carried out on the Origin software platform (OriginLab^®^ Co., Northampton, MA, USA), enabled displaying statistical information with a set of three “summary indices”, referred to as principal components PC1, PC2, and PC3, which clearly differentiated the Raman spectra of different *Candida* species.

A barcode was built with using the deconvoluted Raman spectra of different *Candida* species in order to enable efficient electronic recordkeeping, and increase data accessibility and structural characteristics of different species through apps and user-friendly software. Barcodes could be matched to the Raman spectrum in a number of different ways, and provided the Raman spectroscopic method with the flexibility and the swiftness necessary to inform users about fungal mutations periodically developed under different local conditions. Sequences of Raman Gaussian–Lorentzian bands as deconvoluted from average Raman spectra recorded on different *Candida* species were converted into barcodes by means of an algorithm, which assigned to each band a line with thickness equal to 1/50 of the sub-band width, and a distance from the successive line proportional to the band area.

## 5. Conclusions

The urgent need to unequivocally identify *Candida* pathogen species to enable better risk management for immunocompromised patients calls for the development of novel and more comprehensive diagnostic tools. The present data demonstrated that Raman spectroscopy could represent a breakthrough for early diagnosis of oral fungal infections, while also providing unique insight in terms of structural analysis at the molecular scale. The Raman method showed excellent reproducibility, which underscores the possibility of establishing it to an excellent level of standardization. Although chemometrics could, in principle, remove subjectivity from species determination, the mere morphological nature of these analyses did not enable unequivocal speciation for all four studied oral *Candida* species. Conversely, a newly proposed procedure of Raman barcoding, which directly reflects molecular-scale characteristics, unequivocally located different *Candida* species, in addition to facilitating electronic recordkeeping and empowering it with a rapid access on clouds. Moreover, Raman identification of *Candida* species can be obtained in a few minutes, thus, reducing to a minimum the time necessary for optimizing antifungal treatment decisions.

In conclusion, Raman spectroscopy augmented with the computation of Raman barcodes could reliably be used for recognizing oral *Candida* species. Use of specifically designed Raman instrumentation could allow dental clinics to rapidly identify clinically important *Candida* species in nearly real-time while decreasing analytical costs. More importantly, the availability of a wide library of Raman barcodes for different *Candida* species/clades could enable clinicians to more rapidly make appropriate antifungal choices, decreasing morbidity and mortality in immunocompromised patients. Speciation of *Candida* species by barcode-assisted Raman spectroscopy is based on intrinsic structural differences and offers a rapid, convenient, and reliable method for identification of clinically important oral *Candida* species when compared with cumbersome traditional techniques.

## Data Availability

The data that support the findings of this study are available from the corresponding author upon reasonable request.
